# The Impact of Wheelset Eccentricity on High-Order Polygonal Wear Based on the Theory of Frictional Self-Excited Vibration

**DOI:** 10.3390/ma19101918

**Published:** 2026-05-07

**Authors:** Songhua Zhao, Xiaonan Zhao, Pingping He, Furui Shi, Jie Zheng

**Affiliations:** 1 College of Mechanical and Automotive Engineering, Ningbo University of Technology, Ningbo 315211, China; 13082967256@163.com (S.Z.); z2002120704@outlook.com (P.H.); 2 Department of Mechanical Engineering, University of Albert, Edmonton, AB T6G 2R3, Canadajzheng11@ualberta.ca (J.Z.)

**Keywords:** frictional self-excited vibration, polygonal wear, wheelset–track system, complex eigenvalue analysis, transient dynamic analysis

## Abstract

According to investigation, the wheelset generally appears in a mass eccentric condition. Therefore, the finite element model of a wheelset–track system with mass eccentricity was established in this study to investigate the dynamic response characteristics based on the frictional self-excited vibration theory. The research results show that, when the frictional self-excited vibration of the wheelset–track system occurs, the unstable vibration characteristics of the wheelset–track system corresponding to different dynamic imbalance values are almost the same. That is, the magnitude of the dynamic imbalance value has little influence on the frictional self-excited vibration of the wheelset–track system. Simultaneously, from the perspective of the excitation frequency *f* of the wheel polygonal wear, it shows a trend of increasing frequency with an increase in the running speed. Ultimately, as the phase difference in mass eccentricity grows, pronounced instability becomes evident within the mid- to high-frequency ranges once friction-induced self-excitation arises in the wheelset–track system. This condition readily promotes high-order polygonal wear on the wheel surfaces.

## 1. Introduction

During the operation of high-speed trains, the dynamic balance of wheelsets plays a decisive role in their smooth operation. Wheelset eccentricity is a prevalent mechanical imperfection that can induce additional dynamic loads and exacerbate wheel–rail contact forces, thereby triggering frictional self-excited vibrations in the wheelset–track system. These vibrations accelerate wheel wear and promote the development of wheel polygonal wear and rail corrugation. Furthermore, they produce substantial noise, compromising operational safety. Consequently, a comprehensive investigation of the frictional self-excited vibrations arising from wheelset eccentricity and their underlying dynamic mechanisms is essential to ensure the safe and reliable operation of a high-speed rail system [1,2].

To date, research on the dynamic behavior of wheelsets and the excitation mechanisms underlying frictional self-excited vibrations has been pursued from multiple disciplinary perspectives spanning analytical modeling, experimental testing, and numerical simulation, yielding a well-established and internally consistent theoretical framework. With respect to the research theme, existing studies have predominantly centered on elucidating the initiation mechanisms of frictional self-excited vibrations and their causal relationship with the development of polygonal wheel wear. Study [3] employed the LuGre dynamic friction model to systematically investigate the mechanistic link among lateral self-excited wheel vibrations, Hopf bifurcation phenomena, and the progressive development of polygonal wheel wear. Research in [4] centers on the influence of disc brake dynamics on wheel–rail vibrational interactions, representing the first study to incorporate a high-fidelity disc brake subsystem into a coupled wheel–rail finite element model and to validate the characteristic frequencies associated with high-order polygonal wheel wear through field monitoring data. Authors of [5] developed a vehicle–track coupled dynamic model incorporating high-fidelity wheel–rail friction characteristics and demonstrated that third-order bending resonance of the bogie wheelset assembly constitutes the primary excitation mechanism underlying high-order polygonal wheel wear. Furthermore, the spatial distribution of such wear, specifically its dependence on seasonal environmental conditions and axial position along the wheelset, was systematically characterized. In addition, articles [6,7,8] have demonstrated through various modeling methods that wheelset bending resonance is a key factor contributing to polygonal wear. Articles [9,10,11,12] have, respectively, investigated the influencing factors and suppression methods of polygonal wear from the perspectives of material properties, orbital structure, and system parameters. With respect to the research methods, the combination of numerical simulation and field measurements has become the mainstream paradigm. Study [13] established a rotor dynamics model that incorporates the flexibility and rotational effects of the wheelset and employed the energy method to analyze the stability of the self-excited vibration of the wheelset. Articles [14,15] confirmed the importance of accounting for time-dependent wear in wheel–rail dynamics analysis by integrating field data with multi-body dynamics simulations. With respect to the experimental model, the modeling of a wheelset–track system has evolved from simplified representations to more detailed analyses, shifting from focusing on a single structure to incorporating multiple interacting factors. The two-degree-of-freedom minimal model introduced by [16] provided a concise analytical framework for investigating the self-excited vibrations of tram wheels. Study [17] focused on the phenomenon of parametric resonance in the context of tread taper and revealed the coupling mechanism between mass imbalance and self-excitation. Study [18] established a high-precision dynamic model by the Green function method to analyze the influence of the subgrade system on the wheel polygon wear.

As can be seen from the aforementioned literature, substantial advances have been made in research on the formation mechanism, influencing factors, and modeling methods of frictional self-excited vibration and polygonal wear. However, existing research generally ignores the factor of mass eccentricity, which may occur during operation. Neglecting mass eccentricity may result in an incomplete analysis of formation mechanism and overly optimistic prediction accuracy. Therefore, this study considers mass eccentricity as the entry point to systematically analyze how mass eccentricity causes high-order polygonal wear of wheels by changing the dynamic characteristics of wheelsets.

## 2. Theoretical Framework and Modeling of the Wheelset–Track System

### 2.1. Theory of Frictional Self-Excited Vibration

Frictional self-excited vibration is a self-sustaining oscillatory phenomenon driven by continuous energy input from frictional force. Its core lies in the “speed-dependent” frictional characteristics at the wheel–rail contact interface and the coupling of the system structural dynamics [11,19]. When the wheelset experiences minor disturbances, the friction coefficient decreases with an increase in speed and forms a negative damping effect [19]. This causes the system to continuously draw energy from the rotational motion of the wheelset, thereby inducing unstable vibrations. However, the inclusion of mass eccentricity introduces additional complexities into this mechanism. An uneven mass distribution in the wheelset induces periodic centrifugal disturbances that modulate the wheel–rail normal contact force and generate periodic variations in the frictional force amplitude. These variations, coupled with the negative damping mechanism, ultimately alter the net energy input to the system. This coupling effect directly influences both the stability boundary of self-excited vibration and the characteristics of the resulting vibratory response, thereby establishing a theoretical basis for analyzing how varying degrees of dynamic unbalance, operating speeds, and eccentric phase angles affect the dynamic behavior of the wheel–rail system.

### 2.2. The Characterization of Wheelset Mass Eccentricity

**Figure 1:**
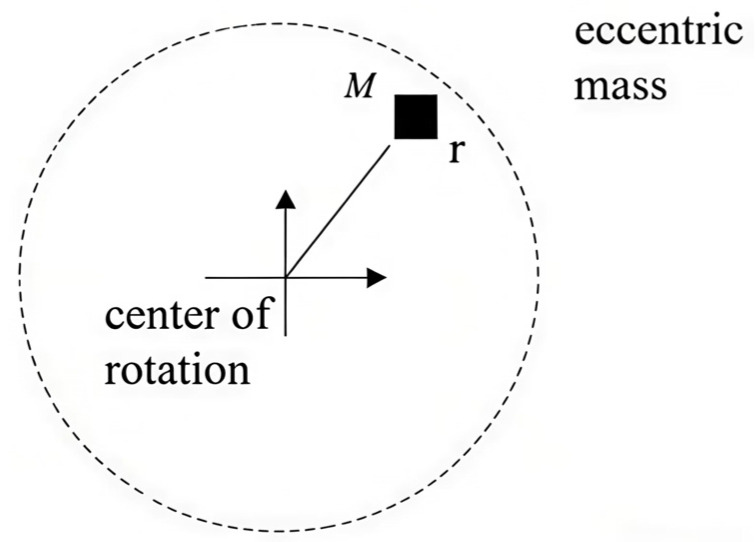
Wheel mass offset.

**Figure 2:**
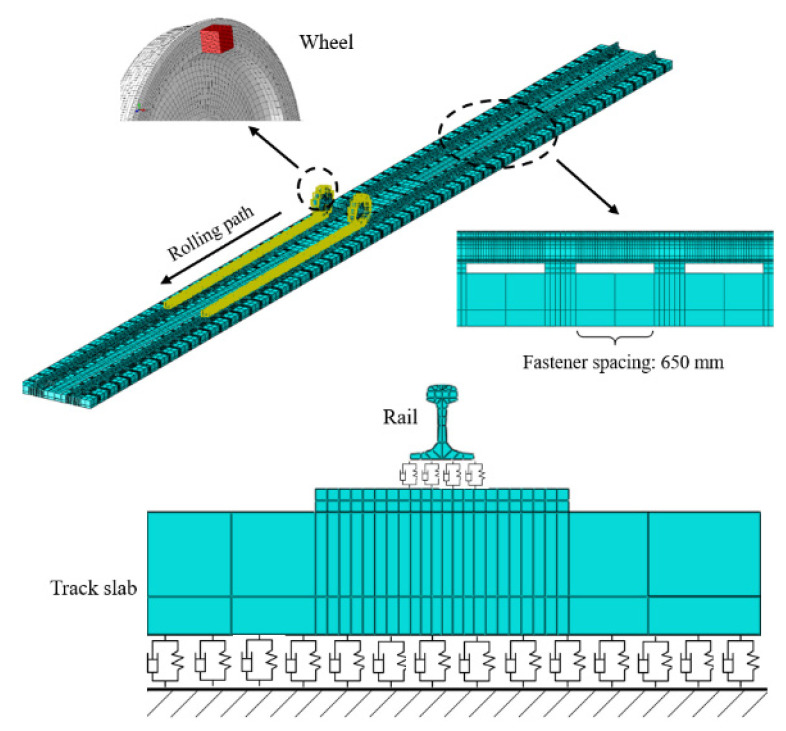
Numerical modeling of the wheel–rail system using finite elements.

Although a wheel’s centroid normally aligns with its rotation axis, manufacturing shape inaccuracies, operational localized abrasion, rust, and additional factors often cause misalignment between the centroid and axis [13]. This is the wheel eccentricity, as shown in Figure 1. The diagram shows the geometric basis for mass imbalance; the wheel’s mass centroid is shifted from its spin axis by a length *R*, with this *R* referred to as the eccentricity. This geometric misalignment gives rise to an unbalanced centrifugal force, which is analytically expressed in Equation (1) and serves as the primary excitation source for all subsequent dynamic analyses. As shown in Figure 2, the finite element model built in this work physically introduces eccentricity *R* by fixing an individual mass piece to the wheelset’s inner wall. The magnitude of the dynamic imbalance *U* was systematically varied by adjusting the material density of the block. Consequently, Figure 1 functions not merely as a conceptual schematic but as a critical conceptual and physical bridge, linking the theoretical formulation in Equation (1) with the numerical implementation, thereby establishing the foundational physical basis for investigating how operating speed, dynamic imbalance magnitude, and eccentric phase difference collectively influence the dynamic response of the wheelset–track system. During the same operation of press-fitting a wheel onto its axle, geometric imperfections in assembly cause an imbalanced dynamic condition in the final wheelset an issue observed in every high-speed railway wheel. During train operation, this situation gives rise to an extra moment of rotational inertia, thereby altering the interaction force at the wheel–rail interface. In these cases, the unbalanced centrifugal force that follows takes the form:


(1) $$F=mR\omega^{2}$$

Here, *R* denotes the off-center radial separation, *m* represents the magnitude of the unbalanced mass, and *ω* signifies the angular velocity of the wheel’s rotation.

### 2.3. Complex Eigenvalue Analysis Methodology

Complex eigenvalue analysis is a fundamental technique for assessing the stability of linear dynamic systems and is particularly suitable for systems with damping, gyroscopic effects, or time delay [20,21]. Its fundamental theory is based on solving the characteristic equation in the state-space form, revealing the vibration characteristics and stability of the system by calculating the complex eigenvalues of the system matrix. Viewed mathematically, Yuan’s [22] model for normal contact constraints adequately represents the frictional coupling at the interface of two elastic bodies. When friction is disregarded, the resulting dynamic equations of the system take the form below:


(2) $$M\ddot{x}+C\dot{x}+Kx=0$$

In this context, the vector representing nodal displacements is denoted by x; *M*, *C*, and *K* denote the symmetric mass, damping, and stiffness matrices, respectively. Consequently, none of the eigenvalues obtained from the characteristic Equation (2) can have a positive real component, indicating that the system is stable. Incorporating the effect of frictional coupling leads to the following expression for the system’s dynamic equation:


(3) $$M_{r}\ddot{x}+C_{r}\dot{x}+K_{r}=0$$

In this expression, the matrices $M_{r}$, $C_{r}$, and $K_{r}$ correspond to the reduced-order approximations of the original mass, damping, and stiffness matrices. Frictional coupling renders all three matrices non-symmetric. Equation (3) gives rise to the following characteristic equation:


(4) $${(M}_{r}\lambda^{2}+C_{r}\lambda +K_{r})\;\emptyset \;=\;0$$

Equation (3) yields the following general solution:


(5) $$x\left(t\right)=\sum \emptyset_{i}exp\lambda_{i}t$$

In this context, $\lambda_{i}=\beta_{i}+j\omega_{i}$ signifies the i-th complex eigenvalue derived from Equation (4), where $\beta_{i}$ and $\omega_{i}$ correspond to the real and imaginary components, respectively, with j denoting the imaginary unit. The symbol $\emptyset_{i}$ represents the eigenvector associated with this same equation. Given that the coefficient matrix in Equation (4) lacks symmetry, eigenvalues possessing positive real components may come into existence. If $\beta_{i}$ (the real part) takes on a positive value, then the system’s nodal displacements grow with time, indicating a progressive reinforcement of vibrational motion and a consequent drift toward instability. Even a minuscule disturbance can cause the oscillation amplitude of the system to enlarge over time. To assess the likelihood of friction-induced self-excited oscillation occurring, one commonly uses the system’s equivalent damping ratio, which is expressed below:


(6) ξi=−βi(π|ωi|)

The negative value of $\xi_{i}$ indicates a propensity for friction-driven self-excited vibration in the system, and this propensity becomes stronger as the absolute value of $\xi_{i}$ increases.

### 2.4. Transient Dynamics Analysis

To examine the system’s unstable oscillations in the time domain, one may apply a nonlinear transient dynamic analysis approach for computing the dynamic response. To achieve full prediction of brake disc vibrations arising from frictional self-excitation during simulation, Nagy [23] constructed a transient dynamic methodology capable of incorporating numerous nonlinearities within the contact model that includes friction. In general, time integration techniques fall into two categories, explicit and implicit, both of which are extensively employed as transient dynamic analysis approaches. The iterative scheme employed to obtain the acceleration solution is what sets the two methods apart. The 2024 version of ABAQUS supports two distinct time integration approaches, each with its own solver. Explicit integration generally uses Abaqus/Explicit, while implicit integration generally uses Abaqus/Standard. From a theoretical standpoint, the solution procedure for the explicit integration employed in this work is briefly outlined below.

The typical approach, founded on explicit transient dynamics, acquires the system’s dynamic behavior via a mass matrix that is diagonally lumped, combined with the central difference technique. The creation of a steady-state system model requires reliance on the relevant temporal steps, listed as:


(7) $${M\ddot{x}}_{\left(t\right)}=P_{\left(t\right)}-I_{\left(t\right)}$$

Within this framework, *M* stands for the aggregated mass matrix, $P_{\left(t\right)}$ is equivalent to the applied external force vector, and $I_{\left(t\right)}$ denotes the system’s internal reaction force vector, with the subscript t designating the specific time instant under consideration. As a result, the acceleration of the system can be formulated as:


(8) $${\ddot{x}}_{\left(t\right)}=M^{-1}(P_{\left(t\right)}-I_{\left(t\right)})$$

Subsequently, the central difference scheme is adopted to carry out explicit time integration. The expressions below yield the system’s nodal velocities at instant (t+∆t) and nodal displacements at instant $\left(t+\frac{∆t}{2}\right)$:


(9) $${\dot{x}}_{\left(t+\frac{∆t}{2}\right)}={\dot{x}}_{\left(t-\frac{∆t}{2}\right)}+\frac{\left({∆t}_{\left(t+∆t\right)}+{∆t}_{\left(t\right)}\right)}{2}{\ddot{x}}_{\left(t\right)}$$

(10) $$x_{\left(t+∆t\right)}=x_{\left(t\right)}+{∆t}_{\left(t+∆t\right)}{\dot{x}}_{\left(t+\frac{∆t}{2}\right)}$$

Here, $\left(t+\frac{∆t}{2}\right)$ and $\left(t-\frac{∆t}{2}\right)$ are subscripts that refer to the midpoints of successive time steps. Under the restriction of the midpoint velocity increment bound, the central difference method cannot be employed. Hence, the starting values for nodal acceleration and velocity need to be specified. Since the system remains stable at the outset, both nodal velocity and acceleration are taken as zero. When advancing to the subsequent increment, the explicit transient dynamic formulation imposes no requirement to verify convergence of the preceding increment. Thus, there is no need to examine whether the system converges while it is running. Nevertheless, for the system to remain stable and for computations to reach a given accuracy level, constructing the time increment ∆t must satisfy the requirements stated below: when no damping is present in the system, the temporal step size must satisfy the following constraint:


(11) $$∆t\leq \frac{2}{\omega_{max}}$$

When damping is present within the system, the time step size can be regulated by employing the set of relations given below:


(12) $$∆t\leq \frac{2}{\omega_{max}}(\sqrt{1+\varepsilon^{2}}-\varepsilon )$$

Here, $\omega_{max}$ represents the maximum natural frequency inherent to the system, while ε corresponds to the critical damping parameter associated with the apex of the modal frequency response. In transient dynamic analysis, the description of contact follows a kinematic approach, whereas friction at the contact interface is governed by a penalty scheme. To conduct a transient dynamic study of the wheelset–track system, the required procedure is as outlined here:

(1) To impose the suspension force on the wheelset, a nonlinear static analysis is conducted on the coupled wheelset–track system.
(2) Nonlinear dynamic evaluation of the wheelset–track assembly aimed at obtaining its transient dynamic behavior.

### 2.5. Finite Element Model of the Wheelset–Track System

**Figure 3:**
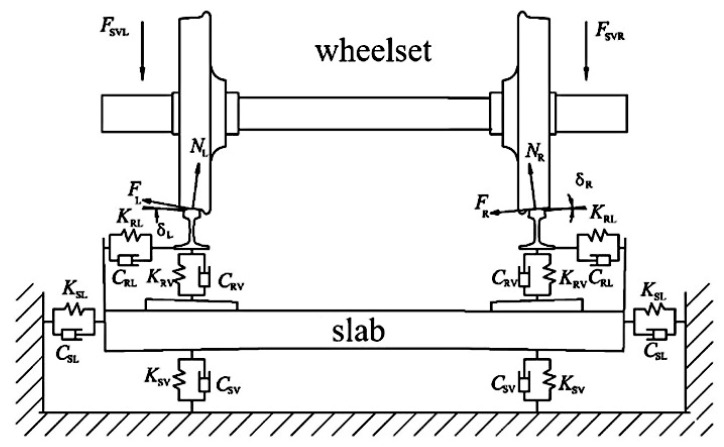
The shape-based interaction taking place on the rail–wheel boundary belonging to the track’s wheel pair assembly.

In this research, an eccentric discrete mass element was affixed to the interior surface of the wheel. The offset radius *R* was maintained constant, while the degree of dynamic imbalance *U* for the wheelset could be adjusted by modifying the value of the eccentric mass *m*. Figure 2 presents a complete finite-element discretization of the wheelset–track system. The mesh for this model consists entirely of C3D8I, a non-conforming element. Contact interfaces between the wheelset and the track are refined through a graded transition mesh, generated using both structured and sweeping techniques. Overall, the model contains 334,583 elements and 1,252,534 nodes. As illustrated in Figure 3, the left and right wheels contact the rails at analogous locations, consistently near the rail tops.

From Figure 3, the vertical forces acting upon the left axle box and right axle box are denoted by *F*_SVL_ and *F*_SVR_, respectively. The quantities *δ_L_* and *δ_R_* represent the contact angles for the left and right, in the same order. At the left and right contact points, the normal forces are *N*_L_ and *N*_R_, respectively, and the creep forces are F_L_ and F_R_, respectively. *K*_RV_ and *K*_RL_ are the vertical and lateral stiffness values of each fastener, respectively. For each fastener, *K*_RV_ and *K*_RL_ stand for the vertical and lateral stiffness coefficients, and *C_RV_* and *C_RL_* similarly represent the damping coefficients in the vertical and lateral directions. *C*_SV_ stands for the vertical component of track-bed support damping and *C_SL_* for the lateral component. Point-to-point spring and damper elements, which possess no mass, connect the rail to the sleeper. Fasteners (tie connections) attach the sleeper to the slab, while massless point-to-ground springs and dampers represent the support provided by the track bed beneath the slab. Table 1 lists the material parameters for each component in the model.

The entire computational procedure consists of two stages. Stage one applies the vertical suspension force as a preload. Stage two establishes the step for explicit transient dynamic analysis. Within the dynamic simulation, the time increment is fixed at 0.0001 s. When recording history outputs, key quantities including the contact force between wheel and rail are extracted. A power spectral density (PSD) analysis is then performed to identify the dominant vibration frequency content of the data. The material properties and the rail-related parameters used in the finite element model for the simulation are listed separately in Table 1 and Table 2.

## 3. Analysis of the Influence of Mass Eccentricity on the Frictional Self-Excited Vibration Characteristics of the Wheelset–Track System

### 3.1. Complex Eigenvalue Analysis

**Figure 4:**
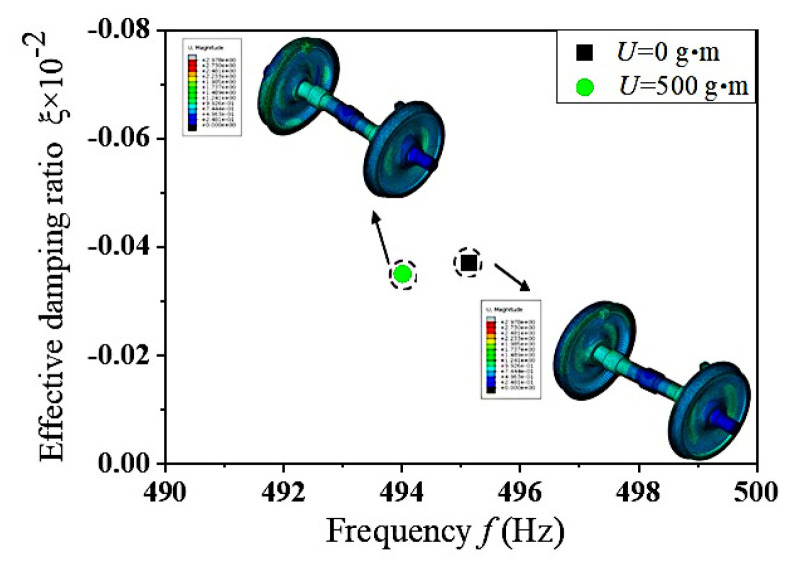
Frequency distribution characteristics and vibration modes of frictional self-excited vibrations in wheelset–track systems.

When high-speed trains equipped with eccentric wheelsets operate on tracks, they frequently undergo acceleration and deceleration through traction and braking systems to adhere to specified speed regulations. Under the impact of traction and braking forces, the longitudinal creep force between the wheelset and rail can reach a saturated state, which may trigger frictional self-excited vibrations within the wheelset–track system. This section carries out a complex eigenvalue analysis under the precondition of defining a value of *U* = 500 g·m and phase difference of 0°. Figure 4 presents the frequency distribution of self-excited vibrations in the wheelset–track system under conditions where the wheel–rail creep forces have reached saturation. For reference, the results obtained from a massless eccentric wheelset are also included for comparison. Under the two operating conditions considered, the frictional self-excited vibrations occur at frequencies of 494.01 Hz and 495.15 Hz, respectively. The corresponding unstable vibration patterns are illustrated in Figure 4, which reveals that unstable vibrations emerge on the wheel tread. To sum up, when frictional self-excited vibrations take place in the wheelset–track system, the impact of wheelset mass eccentricity on its vibration frequency is negligible (with a margin of error below 1%) and can be ignored. For a deeper exploration into how mass eccentricity affects the dynamic response of the wheelset–track system, this research carries out targeted studies from three dimensions, dynamic unbalance, operational speed, and eccentricity phase difference, by means of instantaneous dynamics analysis.

### 3.2. Effects of Mass Eccentricity on Frictional Self-Excitation Vibration of Wheelset and Track Systems

The analysis of complex eigenvalues in the preceding section reveals that mass eccentricity has an insignificant influence on the frequency of friction-induced self-excited vibration in the wheelset–track system. To further investigate its dynamic effects, this study systematically analyzed the impact of the wheelset mass eccentricity on the characteristics of frictional self-excited vibrations in this section based on transient dynamic analysis and by examining three aspects: dynamic imbalance, operating speed, and eccentric phase difference.

#### 3.2.1. Effects of Varying Dynamic Unbalance Magnitudes on Friction-Induced Self-Excited Vibration of Wheelset and Track Systems

**Figure 5:**
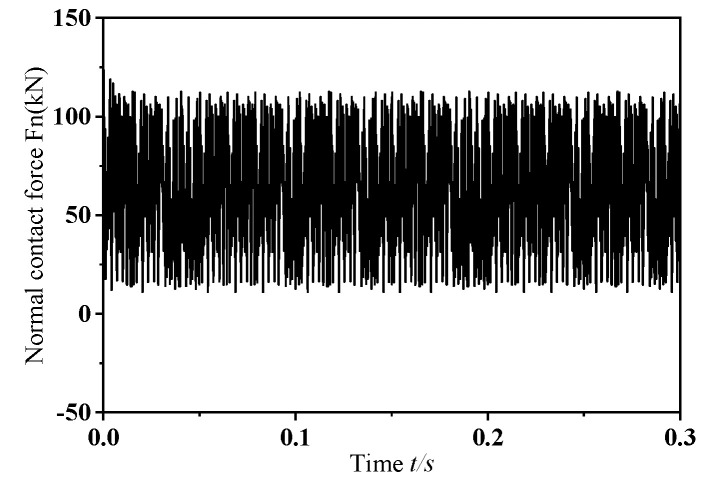
Variation in the normal contact force at the wheel–rail interface under dynamic conditions.

**Figure 6:**
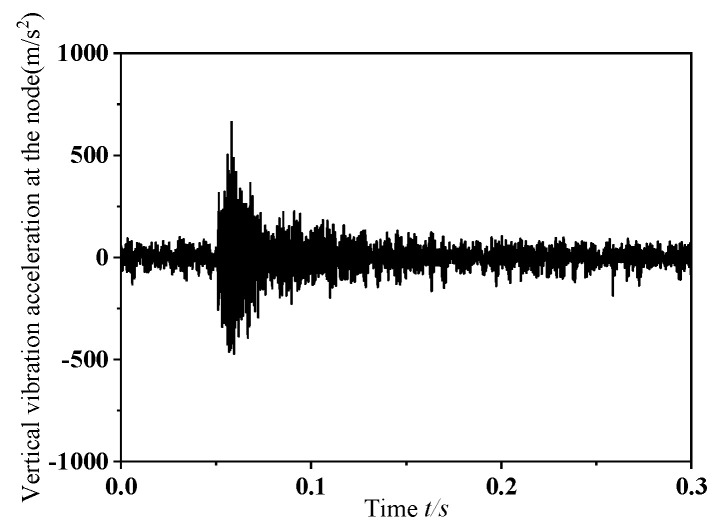
Vertical acceleration response at the designated measurement point.

**Figure 7:**
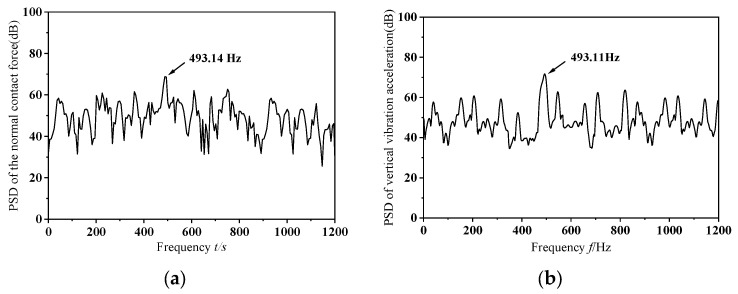
Analysis diagram of vibration signal. (**a**). Normal contact force between wheel and rail. (**b**). Vertical vibration acceleration at the measurement point.

In this section, this study performs transient dynamic simulations on a wheelset that has a dynamic imbalance of *U* = 150 g·m and a zero-degree phase offset in its mass eccentricity, while traveling at a speed of *v* = 300 km/h. The resulting normal contact force between the eccentric-mass wheelset and the rail is depicted in Figure 5, and Figure 6 shows the corresponding changes in vertical vibration acceleration at the selected monitoring point. As illustrated in Figure 5, when the wheelset–track system undergoes frictional self-excited vibration, the normal contact force between the wheel and rail exhibits pronounced fluctuations. As shown in Figure 6, when the wheelset–track system is subjected to frictional self-excited vibration, the vertical acceleration at the measurement point shows marked variations. Furthermore, as the wheel passes the measurement point, the vertical acceleration changes abruptly, with the amplitude increasing instantaneously. The following section presents a power spectral density (PSD) analysis of the extracted normal contact force between the wheel and rail and the vertical acceleration at the measurement point to identify the dominant frequencies causing unstable vibrations in the system during the dynamic wheel–rail contact process. Figure 7 shows the PSD analysis curves for the normal contact force between the wheel and rail and the vertical vibration acceleration at the measurement point. As shown in the figure, the dominant frequencies of the normal contact force and vertical vibration acceleration at the measurement point were 493.14 Hz and 493.11 Hz, respectively. The results of the PSD analysis of the normal contact force between the wheel and rail and the vertical vibration acceleration at the measurement point showed that the dominant frequencies of the unstable vibrations obtained from both were consistent, which aligned with the actual situation. Therefore, it is sufficient to perform PSD analysis only on the extracted normal contact force between the rail and wheel.

**Figure 8:**
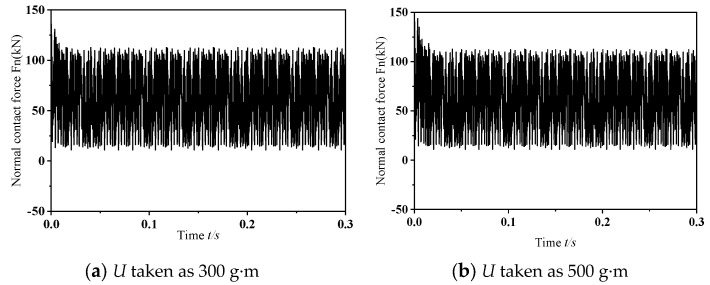
Variation in the normal contact force at the wheel–rail interface under dynamic conditions.

**Figure 9:**
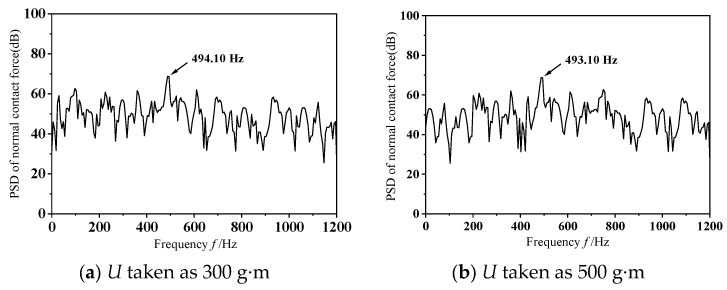
Analysis diagram of vibration signal.

To explore the influence of varying dynamic unbalance masses on frictional self-excited vibrations within the wheelset–track system, this study conducts transient dynamic analyses with *U* = 300 g·m and 500 g·m. Figure 8 depicts the dynamic behavior of the wheel–rail normal contact force under two dynamic imbalance conditions when the wheelset–track system undergoes frictional self-excited vibration. As illustrated in the figure, except for discrepancies in the early stages of frictional self-excited vibration, the vibration features remain largely consistent over time. Figure 9 gives the power spectral density analysis outcomes for the normal contact force at the wheel–rail interface. As shown in the figure, the dominant frequencies of the unstable vibrations in both scenarios are almost identical. In summary, under different levels of dynamic imbalance, the vibration patterns of self-excited vibrations caused by friction in the wheelset–track system are generally similar, indicating that the magnitude of dynamic imbalance has a negligible effect on such vibrations. This is because, during the motion of the wheel set, the excitation of the wheelset–track system caused by dynamic imbalance stems from the impact of the unbalanced centrifugal force generated by the wheelset during high-speed rotation; when wheel slippage takes place, the vibrational behaviors of the wheelset–track system are primarily dominated by friction-driven self-excited vibrations.

#### 3.2.2. Effects of Different Speeds on Friction-Induced Self-Excited Vibration of Wheelset and Track Systems

**Figure 10:**
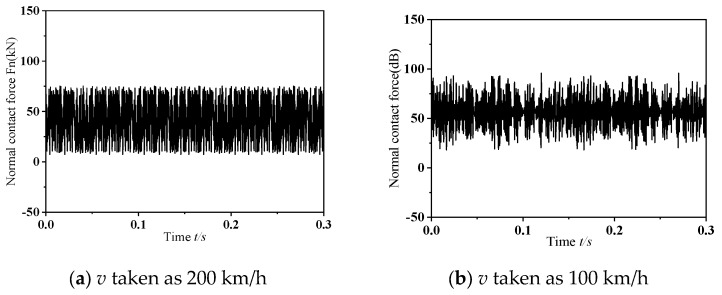
Variation in the normal contact force at the wheel–rail interface under dynamic conditions.

**Figure 11:**
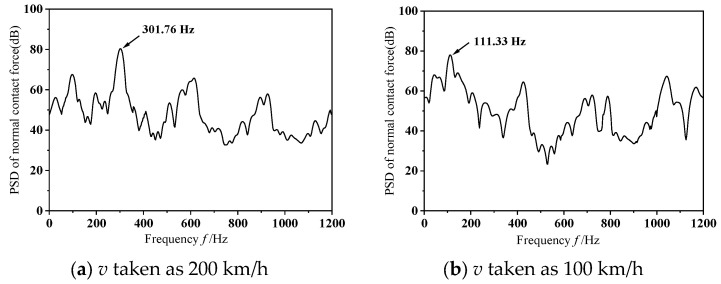
Analysis diagram of vibration signal.

In the analysis of speed-related impacts, this research initially fixes the dynamic imbalance parameter. Specifically, within this segment, an instantaneous dynamic analysis is conducted under the assumptions of a wheelset dynamic imbalance *U* = 150 g·m and the phase difference of 0°. Figure 10 illustrates the dynamic response characteristics of the wheel–rail normal contact force during the occurrence of frictional self-excited vibration in the wheelset–track system at different operating speeds. As depicted in the figure, the normal contact force between wheel and rail shows significant variations, with the peak amplitude of these fluctuations gradually decreasing as the operating speed reduces. Figure 11 shows the outcomes of Power Spectral Density (PSD) analysis applied to the normal contact force at the wheel–rail interface. It can be observed from the figure that, as the speed decreases, the natural frequency of unstable vibrations in the wheelset–track system shows a downward trend. Referring to Figure 7a, when frictional self-excited vibration occurs in the wheelset–track system, the unstable natural frequencies corresponding to the operating speeds of 300 km/h, 200 km/h, and 100 km/h are 493.14 Hz, 301.76 Hz, and 111.33 Hz, respectively. As can be seen from Equation (13) these conditions were likely to cause 17th-order, 15th-order, and 11th-order wheel wear, respectively.

(13) $$f=n\frac{v}{3.6\times 10^{-3}\pi D}$$

where v represents the forward speed, *n* represents the order of the wheel polygon, and *D* represents the wheel diameter.

Overall, when self-excited vibration due to friction occurs within the wheelset–rail system, the excitation frequency corresponding to polygonal wear on the wheels rises with higher operational velocities. As derived from Equation (13), at a fixed speed, a higher unstable vibration frequency corresponds to a higher order of polygonal wear. Consequently, operating speed functions as a critical regulatory factor; by altering the system’s dominant unstable frequency, it dictates the order of polygonal wear and, in doing so, directly impacts the development of high-order polygonal wear under frictional self-excited vibration scenarios.

#### 3.2.3. The Effect of Mass Eccentricity Phase Difference on Friction-Induced Self-Excited Vibration of Wheelset and Track Systems

**Figure 12:**
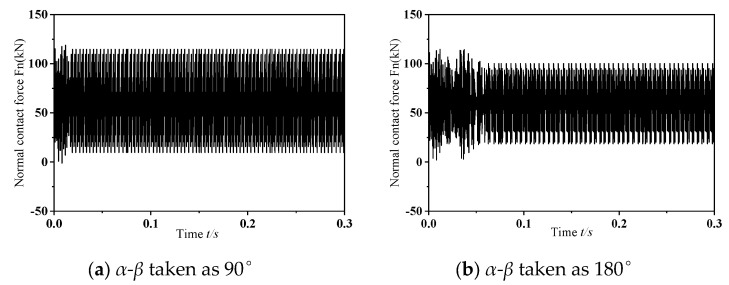
Variation in the normal contact force at the wheel–rail interface under dynamic conditions.

**Figure 13:**
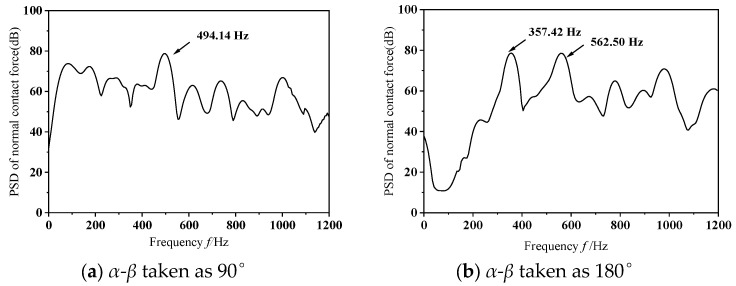
Analysis diagram of vibration signal.

The computational analyses presented in the prior section were predicated on operational scenarios where the phase offset of mass eccentricity was set to 0°. To explore how variations in mass eccentricity phase differences impact frictional self-excited vibrations within the wheelset–track system, subsequent investigations are carried out using phase offsets of 90° and 180°, while maintaining a fixed operational speed of 300 km/h and a consistent dynamic imbalance magnitude of 150 g·m. Figure 12 depicts the dynamic response of the normal contact force at the wheel–rail interface during the occurrence of frictional self-excited vibrations in the wheelset–track system, corresponding to mass eccentricity phase differences of 90° and 180°. The figure indicates that, as the phase difference of mass eccentricity grows, the normal contact force first experiences a short interval of chaotic vibration, then settles into sustained oscillations with a fixed amplitude. This phenomenon arises from the pronounced lateral oscillation of the wheelset around the vertical axis passing through the axle center. Figure 13 depicts the results obtained from PSD analysis of the perpendicular force acting at the interface between wheel and rail. As shown in the figure, when the phase difference between the mass and the eccentricity is 180°, the wheelset–track system exhibits significant unstable vibrations in the mid- to high-frequency range, with a dominant unstable vibration frequency of 562.50 Hz. In summary, an increase in the mass-eccentricity phase difference leads to an increase in the rotational torque. When frictional self-excited vibrations occur in the wheelset–track system, this causes the lateral creep force between the wheel and rail to increase, resulting in more severe contact conditions and making the wheel more prone to polygonal wear. Thus, the phase difference is not only a geometric asymmetry but a critical factor that amplifies lateral dynamic excitation and promotes the occurrence of higher-order polygonal wear under frictional self-excited vibration conditions.

## 4. Discussion

This study found that the magnitude of wheelset dynamic imbalance has a negligible effect on frictional self-excited vibrations. This confirms that the dominant mechanism of self-excited vibrations stems from the coupling between frictional negative damping and structural modes, rather than the intensity of centrifugal excitation. A higher operational speed, however, greatly elevates the frequency of unstable vibrations, thereby producing more pronounced polygonal wear at higher orders on the wheel treads. This finding corroborates the published notion that bending-induced resonance gives rise to higher-order polygonal wear. It is worth noting that an increase in the mass eccentricity phase difference can trigger unstable vibrations in the mid- to high-frequency range (e.g., 562.50 Hz), exacerbate lateral slippage, and worsen contact conditions. This finding expands upon previous research, which viewed eccentricity solely as a source of static imbalance, and reveals that the dynamic torque caused by phase differences is a key underlying factor in the formation of higher-order polygons. In the future, field testing could be combined with multi-body dynamics to further investigate the evolution of eccentric phase differences under complex operating conditions and their quantitative relationship with wheel wear. A limitation of the current model is the linear-elastic material assumption. At high running speeds, the wheel–rail contact pressure may locally approach or exceed the yield strength of the steel. However, the primary focus of this study is the unstable frequency and order of polygonal wear, which are governed by elastic structural dynamics and friction coupling rather than plastic flow. Previous studies on friction-induced vibration in wheel–rail systems have demonstrated that linear-elastic models can reliably predict the onset and dominant frequencies of polygonal wear. Therefore, while absolute wear depths may be underestimated, the conclusions regarding the influence of wheelset eccentricity on the formation of high-order polygonal wear remain valid. Future work should incorporate elastic-plastic material behavior to further refine quantitative predictions.

## 5. Conclusions

From the findings obtained in the present study, the principal conclusions may be stated as follows:

(1) Under different dynamic imbalance conditions, the frequency and amplitude characteristics of frictional self-excited vibrations in the wheelset–track system are virtually identical. This indicates that the vibrations are primarily determined by frictional damping and structural modal coupling rather than the impact of centrifugal force.
(2) When the operating speed rises from 100 km/h to 300 km/h, the natural frequency of unstable vibrations climbs from 111.33 Hz to 493.14 Hz, while the corresponding polygonal wear order of the wheels increases from 11 to 17. This trend demonstrates that running speed acts as a regulatory factor governing the frequency characteristics of high-order polygonal wear.
(3) When the mass eccentricity phase difference between the left and right wheels reaches 180°, the natural frequency of unstable vibration climbs to 562.5 Hz, a condition that readily induces the formation of 19th-order wheel polygonal wear. This indicates that the lateral rocking torque caused by the phase difference is the primary factor contributing to the further deterioration of contact conditions and the occurrence of high-order polygonal wear.

## Figures and Tables

**Table 1:** Each constituent within the finite element model is allocated specific physical property values.

Components	Poisson Ratio	The Young Modulus (GPa)	The Density (kg/m^3^)
Wheelset	0.3	210	7.8 × 10^3^
Axletree	0.29	206	7.8 × 10^3^
Steel rail	0.3	210	7.8 × 10^3^
Rail sleeper	0.2	35	2.5 × 10^3^
Track Slab	0.167	265	1.75 × 10^3^

**Table 2:** Overview of parameters utilized in finite element modeling for railway track configurations.

Name of Parameter	Unit	Representation Symbol	Specific Value
The lateral inclination of the rail	–	*δ*	1/40
Friction factor of wheel-rail contact surface	–	*μ*	0.45
Center distance of sleepers	mm	*l*_s_	650
Diameter of the rolling circle of the wheel	mm	d	920
Lateral damping of the fastener system	Ns/m	*C*_RL1_	1830.22
Vertical damping of the fastener system	kNs/m	*C*_RV_	20
Longitudinal damping of the fastener system	Ns/m	*C*_RL2_	1830.22
Lateral stiffness of the fastener system	kN/mm	*K*_RL1_	9
Vertical stiffness of the fastener system	kN/mm	*K*_RV_	50
Longitudinal stiffness of the fastener system	kN/mm	*K*_RL2_	9
Lateral support damping of the track plate	kNs/m	*C*_SL1_	40
Vertical support damping of the track plate	kNs/m	*C*_SV_	310
Longitudinal support damping of the track plate	kNs/m	*C*_SL2_	40
Lateral support stiffness of the track plate	kN/mm	*K*_SL1_	50
Vertical support stiffness of the track plate	kN/mm	*K*_SV_	170
Longitudinal support stiffness of the track plate	kN/mm	*K*_SL2_	50
Vertical suspension load on the left side	N	*F*_SVL_	60,000
Vertical suspension load on the right side	N	*F*_SVR_	60,000

## Data Availability

The original contributions presented in this study are included in the article. Further inquiries can be directed to the corresponding author.
